# Nanometric depth resolution from multi-focal images in microscopy

**DOI:** 10.1098/rsif.2010.0508

**Published:** 2011-01-19

**Authors:** Heather I. C. Dalgarno, Paul A. Dalgarno, Adetunmise C. Dada, Catherine E. Towers, Gavin J. Gibson, Richard M. Parton, Ilan Davis, Richard J. Warburton, Alan H. Greenaway

**Affiliations:** 1 Physics, SUPA/IIS, School of Engineering and Physical Sciences, Heriot-Watt University, Edinburgh EH14 4AS, UK; 2 School of Mathematical and Computer Sciences, Heriot-Watt University, Edinburgh EH14 4AS, UK; 3 School of Mechanical Engineering, University of Leeds, Leeds LS2 9JT, UK; 4 Department of Biochemistry, University of Oxford, Oxford OX1 3QU, UK; 5 Department of Physics, University of Basel, Klingelbergstrasse 82, 4056 Basel, Switzerland

**Keywords:** particle tracking, three-dimensional imaging, photo-activated localization microscopy, micro-fluid flow, image sharpness, maximum likelihood

## Abstract

We describe a method for tracking the position of small features in three dimensions from images recorded on a standard microscope with an inexpensive attachment between the microscope and the camera. The depth-measurement accuracy of this method is tested experimentally on a wide-field, inverted microscope and is shown to give approximately 8 nm depth resolution, over a specimen depth of approximately 6 µm, when using a 12-bit charge-coupled device (CCD) camera and very bright but unresolved particles. To assess low-flux limitations a theoretical model is used to derive an analytical expression for the minimum variance bound. The approximations used in the analytical treatment are tested using numerical simulations. It is concluded that approximately 14 nm depth resolution is achievable with flux levels available when tracking fluorescent sources in three dimensions in live-cell biology and that the method is suitable for three-dimensional photo-activated localization microscopy resolution. Sub-nanometre resolution could be achieved with photon-counting techniques at high flux levels.

## 1. Introduction

Estimating particle position and/or motion from images or time-lapse image sequences is widely used in biology for imaging proteins [1–3] or live cells [3–5] and in fluid-flow applications [6,7]. In live-cell imaging, the need to minimize photo-damage to the specimen leads to a requirement for efficient flux collection, and thus to the use of high numerical aperture (NA) objectives, with consequently shallow depth of field. When combined with wide-field imaging requirements, this approach leads to high-quality, in-focus images only over a relatively thin specimen layer. Layers above and below this in-focus specimen slice are defocused, and thus recorded with a blurred point-spread function (PSF). The reduced contrast in these out-of-focus images leads to poorer signal to noise and to an impaired capability to track particles having out-of-plane motion within the specimen.

Approaches used to recover this lost out-of-plane tracking include a through-focal series or *z*-stack [8], modelling the out-of-focus image in order to estimate the depth of point sources [9], the use of multiple objectives imaging the specimen simultaneously from both above and below [10–12] or using two different-focus images obtained by incorporating a beam splitter within the imaging system [2,3,13–16], parallax [17], scanning [18], anamorphic imaging [13,19,20], the unique double-helix PSF [21] and holography, in-line [22] or off-axis [23].

The *z*-stack has the principal disadvantage that it is time consuming, and the live specimen changes during the recording sequence, leading to a loss of information concerning rapid motion and different fluorophore-bleaching or quantum-dot blinking effects in each image. The image-modelling approach requires a good knowledge of the optical system and has poor sensitivity close to in-focus image conditions. The multiple-objective approach has the advantage that flux emitted by the specimen in two or more directions can be collected and imaged, but the images obtained may need to be inverted, rotated and scaled in both intensity and magnification before analysis of the fused dataset can be robust and efficient. Interferometric-based techniques using opposing objectives [11,12] are susceptible to vibration-induced errors typical of non-common path optics, and the novel objective and specimen mounting may inhibit use under standard microscopy laboratory conditions. Scanning techniques can achieve a good resolution while limiting specimen photo-damage, but, in wide-field applications, the scanning sequence required to deliver three-dimensional information can take longer than a *z*-stack sequence, although localized imaging could be achieved at high speed. A holographic approach can compromise real-time image interpretation.

Here, we use a diffractive optical element (DOE) to achieve simultaneous in-focus images of multiple specimen layers, recorded through a single objective lens, at equal magnifications, on a single camera and with fixed position registration between the in-focus layers. The simple and inexpensive attachment required can be interposed between a standard microscope and the user's favourite camera, and can be used reliably under standard laboratory conditions (e.g. no temperature control, no vibration isolation, room lights on). We show that using these multi-focal images with a simple metric (image sharpness) and a maximum-likelihood algorithm gives robust depth determination at nanometric resolution when applied to images of unresolved sources that are not background limited (i.e. good contrast).

The method is intended for three-dimensional tracking of faint sources in biology. In this paper, we will describe our methodology and show, using experimental data, simulated data and analytical methods, how our multi-focal image-sharpness approach delivers very high depth resolution with potentially real-time results. The experimental measurements are confined to bright sources, and show that the previous table-top optics [24] can be reduced to a modest-sized and low-cost microscope attachment without sacrificing the particle-tracking accuracy. Like Aguet *et al*. [9], we use a maximum-likelihood estimation and develop an analytical formula that can be used to assess likely performance for this method at flux levels that are more appropriate to biological measurements, and test that analytical formulation through numerical simulation.

## 2. Implementation on a standard microscope (olympus ix71)

The DOEs and the optical system used to achieve simultaneous in-focus images of multiple *z*-planes have been described before [25], as have the modifications required to make the system work under ‘white light’ conditions [26] and to ensure that the images of all *z*-planes have equal magnification and well-defined spacing of the in-focus planes [27]. The resulting microscope attachment has been used experimentally on both upright and inverted commercial microscope systems, with a range of objective lenses, to deliver between two and nine simultaneous, in-focus image planes on a single charge-coupled device (CCD) camera. In this paper, a microscope attachment delivering three in-focus image planes, to a single CCD and on an inverted microscope, is intended unless otherwise stated.

**Figure 1.**
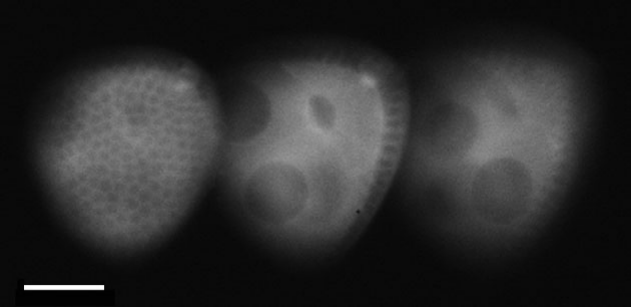
A fly (*Drosophila melanogaster*) egg chamber expressing a green fluorescence-tagged protein and imaged using epifluorescence, a 0.7NA dry 20× objective and the DOE-based attachment with 7.3 µm separation between the in-focus planes. Left to right the three images show: the surface of the egg chamber within the egg chamber; the far side of the oocyte and nurse cells deeper within the egg chamber. Scale bar, 20 µm.

Briefly, a DOE in the form of an off-axis Fresnel lens has a different focal length in each diffraction order. If this DOE is combined carefully with a lens of high optical power, the system focal length in each diffraction order can be arranged to deliver in-focus and spatially separated images in which the image in each diffraction order is equivalent to an image that could have been recorded in a *z*-stack sequence. The etch depth (thus phase modulation) of the DOE determines the relative brightness of the images in the various diffraction orders. The lateral resolution, depth of focus and depth of field of the image in each diffraction order is equal to the performance that would have been achieved from the equivalent image in a sequential *z*-stack. Used as a simple three-dimensional snapshot system, the technique delivers images of the sort shown in figure 1; see also [28]. The DOE combines the function of the beamsplitter and defocus lens in other multi-focus techniques (e.g. [16]).

**Figure 2.**
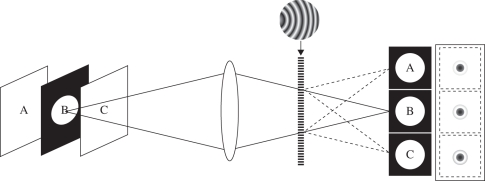
Schematic of the DOE-based three-dimensional imaging attachment. An off-axis Fresnel lens positioned at a distance of one focal length from the secondary principal plane of an imaging system produces three images, each focused on a different specimen plane and all recorded with equal magnification. The in-focus plane separation increases with increasing curvature of the lines in the DOE. Crossing two such gratings delivers nine different in-focus *z*-planes. Inserts show the DOE structure and images of a nano-hole from three DOE diffraction orders (inverted contrast and saturated to show image structure when the nano-hole is positioned well away from focus, at *z* = −2.1 µm in figure 3*a*). The schematic represents a unit-magnification relay system attached to the microscope camera port. The microscope camera would normally be located at the position of the letter B on the left-hand side of the figure. An aperture or slit is located at B to prevent overlap of the images of the different *z*-planes on the camera, which is now located on the right-hand side.

For three-dimensional tracking tests, we used a nano-hole, illuminated from above with a laser, to represent a single unresolved and self-luminous ‘particle’. This particle, imaged through the microscope plus the DOE system described above, yields three images, each corresponding to a PSF that would have been recorded in a *z*-stack sequence and with about one-third of the detected flux in each image. If the total measurement time is equal to that required for the equivalent three-image *z*-stack, the flux in each of the three images is equal whether the DOE system or a *z*-stack is used. This system is illustrated schematically in figure 2, together with example out-of-focus particle images from a simultaneous three-image snapshot. The snapshot was recorded using a 100 × 1.4NA oil-immersion objective lens (UPLSAPO100XO/1.4), with an off-axis Fresnel lens of focal length 0.94 m in a unit magnification optical relay from the microscope focus to the CCD camera employing an achromatic compound lens of focal length 76.2 mm. The source is a nominally 210 nm diameter hole in an NiCr/Al/NiCr film approximately 90 nm thick, illuminated by a laser at 532 nm wavelength and mounted on a precision Mad City Labs (NanoView PDQ375/M) translation stage that provides *z*-displacement of the source under computer control (Micromanager) with sub-nanometre level accuracy and repeatability.

The microscope was focused to give a best focus in the in-focus zero-order (i.e. central) image and the exposure adjusted to give a peak of approximately 75 per cent of the 12-bit dynamic range available from the Qimaging Retiga SRV CCD camera. Using the camera's QCapturePro software, a series of 50 images was recorded at each of 100 computer-controlled *z*-positions with nominally equal source flux and unit camera gain. A total of eight separate datasets were recorded over an eight-day elapse period. For each dataset described here, the acquisition procedure was: (i) reset focus (by eye) in the zero-order image at the centre of the translation range, (ii) translate the source to the minimum *z*-position, and (iii) to scan in *z* recording all 50 data frames at each *z*-position before moving to the next *z*-position. Examination of the recorded *z*-scan data shows that the position of best focus in the zero order (determined by peak sharpness) varies by ±1 µm from the nominal position. Drifts in laser brightness during measurements meant that two sets contained saturated images and were rejected. Thus, all images analysed are unsaturated under the imaging conditions used. The 6.45 µm CCD pixel spacing provides a slight oversampling of the images. Assessment of the in-focus images obtained when the source is translated in *z* revealed no significant degradation of the in-focus image quality between the DOE diffraction orders [24].

The algorithm used here exploits image sharpness [29], a measurement originally proposed for high-resolution astronomy, but abandoned in that application as it provides a metric reaching a global maximum when diffraction-limited imaging conditions prevail but does not indicate what corrections to the optical system will achieve that optimum.

The image sharpness is the integral of the square of the image intensity. Experimentally, each CCD data frame from our microscope attachment contains three images of the same particles. From each data frame three normalized image sharpness values, *S*, are evaluated by selecting three subregions of the same shape and roughly centred on each of the three particle images. The subregion size is chosen to include all significant flux from the particle studied, but to exclude flux from neighbouring particles. For each of these three images of the particle, the pixel values are background subtracted, squared, summed over the subregion and divided by the square of the total flux in the appropriate subregion, giving three flux-independent, normalized, sharpness-based measures of image quality. Here *S* is evaluated *a posteriori* using digitized CCD frames that have been stored in a computer, but it is noted that using complementary metal oxide semiconductor (CMOS) detector technology would allow *S* to be calculated on-chip and, with suitable windowing and calibration, delivered as the detector readout in real time.

## 3. The maximum-likelihood sharpness algorithm

For a given source position, *z*, a set of repeated *S*-measurements made in each diffraction order can be used as calibration data to estimate probability density functions (PDFs) for the experimental sharpness measurements in each diffraction order when the source is at *z*. These experimentally determined PDFs subsume all errors owing to photon flux, background noise, instrumental drift, optical defects, etc., over the duration of the calibration measurements. If the diffraction orders are denoted using the subscript *j*, we can express the probability for any given *S* measurement in the form of a conditional probability *P*(*S*_*j*_|*z*). With *z* given, the sharpness estimations in the different diffraction orders are independent, so the probability for the subset of *M* sharpness measurements in all diffraction orders when a particle is located at *z* may be expressed3.1
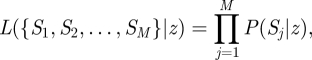
where *L* is the likelihood for the subset of sharpness measurements {*S*_1_, *S*_2_, … , *S*_*M*_} and *M* is the number of diffraction orders in which images are simultaneously recorded. The maximum-likelihood (ML) estimator of the source depth, 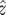, is the value of *z* for which *L* is maximized given an actual data subset {*S*_1_, *S*_2_, …, *S*_*M*_} and the calibration PDFs, *P*(*S*_*j*_|*z*).

To estimate *P*(*S*_*j*_|*z*) from calibration data, curves were fitted to the measured mean sharpness value, 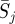, and the variance on the sharpness at each calibration *z*-point. For 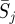 a cubic-spline fit to the actual data at all *z* was used, so this fit subsumes all errors owing to spherical aberration or other optical defects. In the case of the variance on the sharpness, a Gaussian fit to the measured variance over the *z*-range was used to smooth the results and provide a calibration variance. These fits were used to interpolate the calibration parameters between the *z*-values at which the calibration measurements were made. For reduction of the experimental data, *P*(*S*_*j*_|*z*) was assumed to be Gaussian at any *z*, with mean 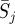 determined from the cubic-spline fit and variance determined by the Gaussian fit to the measured variance values. Note that the variance is flux dependent and needs either to be estimated from images at several flux levels or adjusted using the Poisson flux statistics. Details of the method for estimating *P*(*S*_*j*_|*z*) from calibration data are provided in the electronic supplementary material.

## 4. Experimental test results

**Figure 3.**
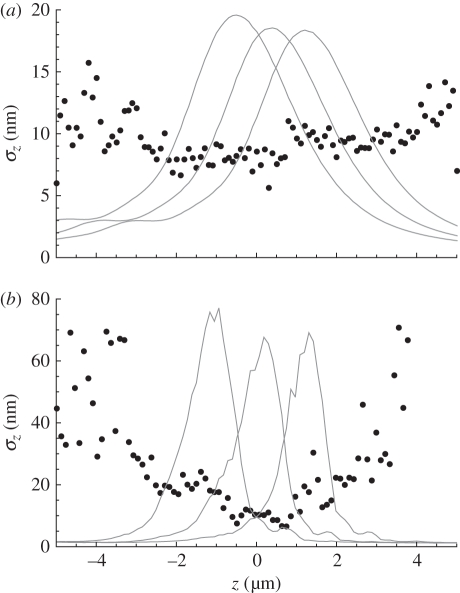
Root-mean-square error (points) in ML point-source depth estimation, using the standard deviation between the ML estimate of *z* and taking the translation stage position (*z*) as ground truth. The points thus show the depth-measurement accuracy achieved. The curves show experimental sharpness-calibration measurements in each of the three images (arb. units), (*a*) data recorded using an Olympus IX71, (*b*) data recorded using our optical-bench assembled microscope [24]. Particularly in (*b*) note that the accuracy is generally lower for *z*-values corresponding to the sharpness peak in any diffraction order and that the accuracy decreases for *z*-values significantly outside the volume between the extreme in-focus image planes. Objective NA was 1.4 for (*a*) and 1.3 for (*b*). The wider sharpness curves in (*a*) appear to be due to directional ‘beaming’ from the nano-hole source, leading to underfilling the objective. The sample was damaged while trying to make AFM measurements to verify that conclusion.

The source *z*-position is estimated from the measured sharpness values using an ML algorithm. Figure 3 shows the experimentally determined standard deviation for the ML-estimated *z*-position of a source using a dataset obtained from the IX71 (figure 3*a*) and earlier data from an optical-bench assembled ‘microscope’ (figure 3*b*) [24]. In the background, in each plot, the calibration curves of *S* versus source *z*-position for each diffraction order are shown (the position indicated by the translation stage is taken as ‘ground truth’). Each curve represents an average of 50 measurements at each of 100 source *z*-positions. Each complete 5000-image dataset took about 1 h to acquire on the IX71. Neither temperature-control nor vibration-isolation precautions were implemented. The microscope focus relies on the standard mechanical and unstabilized objective-focus control. Thus, we believe that such measurements are achievable in almost any laboratory.

The peak value for *S* in each diffraction order occurs close to the position in which the source should be in focus in that diffraction order (allowing for aberrations and refractive indices), and decreases as the source position moves away from this location in either direction. Thus, the measurement of *S* in a single image plane yields the source *z*-position to within a twofold ambiguity. It is clear that, under the imaging conditions used here, all *S*-curves show only a single maximum within the depth range of interest. Thus, the simultaneous measurement of *S* under two or more different focal conditions provides a unique determination of the source *z*-position. Because the calibration and data inversion use the same optics, neither the *z*-spacing between the three image planes nor the optical efficiency associated with the diffraction orders needs to be precisely known, an advantage when using real systems.

The analysis of experimental data with *M* = 3 demonstrates between 7 and 14 nm level accuracy across the five unsaturated datasets available; see example in figure 3*a*. Different data recorded 12 months earlier on our optical-bench-assembled microscope (figure 3*b*) yielded 12 nm r.m.s. depth resolution [24], even though those data suffered significant spherical aberration, suggesting that approximately 10 nm is a realistic accuracy limit for high-flux measurements using a CCD with 12-bit well depth.

We find that datasets recorded at different times can give 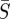-curves that differ by more than the standard deviations within individual datasets and attribute this to focus drift, because returning the *z*-stage to the start position did not give good focus in the particle images in the zero diffraction order. Small amounts of drift can affect calibration and, to identify circumstances where such instabilities degrade the reliability of the *z* determinations, the ML algorithm reports any data frames for which the ML-estimated *z* corresponds to a likelihood significantly lower than average, so that such data can be examined in detail. We found that this alarm successfully identified suspect calibrations and that, if one does not wish to repeat the calibration, increasing the estimated variance would eliminate the alarm at the penalty of a modest increase in the uncertainty of the depth measurements.

## 5. Analysis of the maximum-likelihood algorithm

Having established experimentally that the ML-sharpness algorithm can deliver accurate source-position estimations that are easily computed from the high-flux three-dimensional snapshot images captured using the DOE-based imaging system, it is interesting to consider how the precision achieved will depend on the details of the experimental situation, especially source flux available, number and separation of diffraction orders used in the dataset, detector efficiency, etc.

This section will develop an analytical solution for the minimum variance bound (MVB [30]; also known as the Cramer–Rao lower bound) in order to assess the accuracy obtainable in a particular experimental situation and thus to optimize instrument design for particular measurement requirements. We will also consider how to scale the calibration variance to allow for different particle fluxes when estimating *P*(*S*_*j*_|*z*).

We assume that the DOE introduces no differential aberrations that affect image formation in different diffraction orders, justified on the basis of earlier experimental results [24], and model all aberrations and apertures affecting the images as cylindrically symmetric with respect to the optical axes. Thus, a theoretical image sharpness, 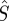, may be expressed using the well-known Fourier–Bessel transform to exploit the cylindrical symmetry and reduce the integral to one dimension5.1
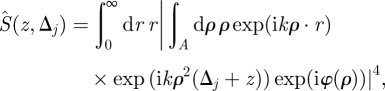
where *r* is the radial distance in the image plane measured from the axial position associated with the *j*th DOE diffraction order, *ρ* is the radial coordinate in the objective lens pupil plane measured from the optical axis, *Δ*_*j*_ is the DOE-introduced focus change in the *j*th diffraction order and expressed in wavelength units, *z* is the focus change measured in waves, induced by the particle axial position relative to a reference plane in which *Δ* = 0 (assuming, without loss of generality, that *Δ*_0_ = 0), *k* = 2*π*/*λ*, *φ*(*ρ*) represents other optical defects (e.g. spherical aberration) and *A* is the disc |*ρ*|^2^ ≤ 1, restricting the area of integration to the transparent region of the lens pupil. Thus, a focus change *Δ* = 1 corresponds to one wave of sag in the objective pupil and *z* = 1 corresponds to an axial source displacement of 2*λ*/*NA*^2^ (or four times depth of field). Numerical evaluation of equation (5.1) indicates that 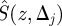 falls to half its peak value for *Δ*_*j*_ + *z* ∼ 0.34, showing that sharpness is a sensitive indicator of focus.

For any estimator, the MVB provides a greatest lower bound for the variance of the estimation, irrespective of the algorithm used to invert the data. Thus, the MVB provides a measure of the ability of the data model to discriminate between different values of the parameter to be estimated from data described by that model.

The MVB for our problem can be expressed5.2
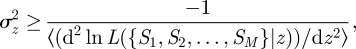
where the ensemble average is to be taken over data subsets {*S*_1_, *S*_2_, … , *S*_*M*_} folding in all statistical processes (notably the photon-counting statistics associated with the imaging process considered here) that need to be included in the description of the signal detection and processing. The data vector {*S*_1_, *S*_2_, … , *S*_*M*_} represents all of the information extracted from the data that will be used in data inversion. Equation (5.2) thus represents the sensitivity of the data {*S*_1_, *S*_2_, … , *S*_*M*_} to the particle depth—i.e. to the parameter to be estimated.

When using an idealized photon-counting detector, the detection model is a Poisson process where *μ*_*k*_, the intensity in detector pixel *k*, may be expressed as the mean photon count rate per data frame. Under these circumstances, it is known [31] that the value of *S* determined by squaring and summing the detector output is biased but that the bias is easily eliminated, while improving the signal to noise, by subtracting the photon count in each pixel from the square of that count. This bias subtraction ensures that only pixels containing two or more photons contribute to the sharpness (otherwise the sharpness has a floor determined by the mean photon count in the image, irrespective of the severity of aberration).

In our system, the DOE acts as both a beam splitter and a focus-changing element, and the relative strength and number of diffraction orders produced (thus number of in-focus planes) depend on the DOE structure. Our DOEs have a single etch depth (thus phase modulation) in a fused-silica substrate, selected such that the energy diffracted into the 0 and ±1 diffraction orders is balanced and equal to approximately 28 per cent of the incident flux (the remaining 16% is lost into higher diffraction orders). Thus, approximately 84 per cent of the incident flux is available in the three diffraction orders used in data analysis. Higher photometric efficiency is achievable through the use of more than a single etch depth (thus for greater expense), and hereafter it will be assumed that the source flux is divided between all diffraction orders without loss.

Under these assumptions, each diffraction order contains *M*^−1^ of the source flux collected by the objective lens. For a Poisson detection process and pixel means *μ*_*k*_, the ensemble-average sharpness from summing the pixel count squared minus the pixel count provides an unbiased estimate of the sharpness associated with the mean image intensity. The variance on the measured *S* is found to be ([31]; see also the electronic supplementary material)5.3
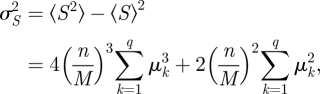
where *n* is the total detected flux in all *M* images and *q* is the number of detector pixels in each image. The DOE reduces the flux, and thus the signal-to-noise ratio (SNR) of the sharpness measurement per image, but increases the number of images available for measurement, each giving a different *S* curve as a function of *z* (see background curves in figure 3). When the flux per image, *n/M*, is large, the leading term will dominate in equation (5.3). However, the image intensity is normalized, 
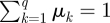
, so *μ*_*k*_ ≤ 1 ∀ *k* and 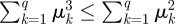. In the low-flux regime, where
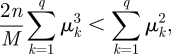
the second term will dominate. The flux at which the contribution from these two terms matches is dependent on the image intensity distribution—at any flux level, there will always be a degree of image defocus beyond which the second term will dominate. From numerical evaluation of equation (5.3) we find that, in the regimes of interest here, for images suffering only defocus aberration, the leading term dominates when more than about 100 photons per image are recorded.

Numerical solution of equations (5.1) and (5.3) suggests that, over the depth ranges of interest and at low flux, both *S* and 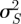 can be modelled using suitably weighted Lorentzian functions with the same NA-dependent half-width, *a*, corresponding to a defocus-induced change of 0.34 *λ* in wavefront shape across the objective lens. This is reasonably consistent with our experimental data. For low flux, we model the PDF for any given sharpness measurement as a gamma distribution having a mean determined from equation (5.1) and a shape parameter chosen to give a variance according to the leading term in equation (5.3) (see electronic supplementary material). The integrations involved in equation (5.2) then become tractable (in Mathematica) and the MVB may be expressed, for *n* detected photons in *M* in-focus image planes separated by *δ* as5.4
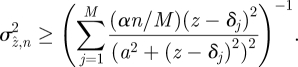


The assumption of gamma-distributed statistics ensures that the modelled sharpness never takes physically impossible negative values. The constant, *α*, estimated from numerical evaluation of 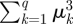 has a value of approximately 2.9. A Gaussian, rather than a gamma distribution, was used in the reduction of experimental data. A Gaussian model leads to an expression equivalent to equation (5.4) with *α* ⇒1 for *n*/*M* ≫ 1, but implies physically impossible negative sharpness values, albeit with low probability. The central-limit theorem suggests that a Gaussian PDF will be valid at high flux, but here we are principally interested in photon-counting measurements at modest flux levels and an intermediate value, *α* = 2, is adopted hereafter.

Equation (5.4) may be used for experiment design to determine, for a given particle flux *n* per data frame and objective described by *a* (the *z*-shift that produces a 0.34 *λ* change in curvature of the spherical wavefront across the objective aperture), the optimum choices of *M* and *δ*, the parameters that determine the DOE design. The derivation of equation (5.4) involves some heuristically justified assumptions (e.g. use of the gamma distribution, use of a Lorentzian shape function), so comparison with experiment, simulation and prior work is useful to provide confidence in its application.

Firstly, if a single image plane is chosen (*M* = 1), the variance on the *z*-measurement becomes infinite (is singular) when the particle is in focus, *z* = *δ*_1_. This is consistent with prior work, where the source depth is estimated using a parametric fit to the image PSF [9,15]. In essence, the turning point at maximum sharpness, when the image is in focus, provides no depth sensitivity in the measurement. Even away from focus, a single sharpness measure gives a twofold ambiguity in depth estimation unless it is known *a priori* that the best focus is outside the sample volume.

**Figure 4.**
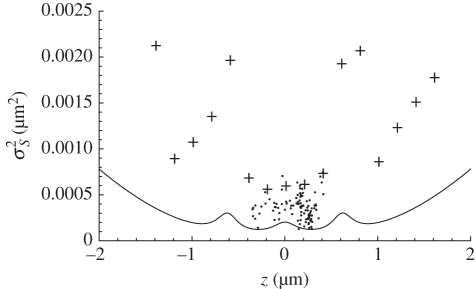
The line shows the MVB for sharpness ranging on a point source emitting an average of 768 detected photons distributed between the DOE diffraction orders and evaluated using equation (5.4) with *α* = 2. The axis labels in micrometres are correct for an objective of NA = 1.26 and wavelength 500 nm. The spacing of the in-focus planes is 0.6 µm and the Lorentzian half-width *a* = 0.334 µm. The MVB suggests that for the specimen volume between the outermost in-focus planes a measurement uncertainty of approximately 14 nm is obtainable with this level of detected flux. The local peaks in measurement uncertainty correspond accurately with the positions of the in-focus planes. The accuracy will scale inversely with the square of the numerical aperture, so the flux requirements will fall by about 20% for NA = 1.4. The crosses and dots represent r.m.s. errors (as in figure 3) for results from numerical simulations; the dots are taken from the movie data shown in figure 5.

Using more than one in-focus plane resolves the singularity and provides a unique estimation of the depth of the source. Figure 4 shows the MVB estimated from equation (5.4) for three in-focus image planes (*M* = 3), *α* = 2 and mean flux of 768 photons (approx. 256 in each image). Note that the curve in figure 4 shows qualitative agreement with the high-flux experimental results in figure 3, in particular the variance minima correspond with the cross-over points in the sharpness curves and the local variance maxima correspond to the positions of the in-focus image planes.

Interestingly, provided that one restricts *δ* ≈ 2*a* (thus separation between the in-focus planes is comparable to the sharpness full-width half maximum), the mean MVB as expressed by equation (5.4) for *z*-values within the volume enclosed by the extreme in-focus planes is almost independent of the number of images used. This result may be understood by considering that, although the SNR on each individual sharpness value scales as (*n*/*M*)^0.5^, the number of independent sharpness estimates increases at a rate that compensates for that fall in SNR, provided that all measurements remain within the high-flux regime (more than 100 photons per image). As the available photon flux falls, reducing the image-plane separation to *δ* approximately *a*, i.e. close to the depth of field in value, helps to concentrate the available photons and thus to maintain depth-measurement accuracy at the expense of the total depth over which the measurements can be made. Reducing the image-plane separation further offers no useful gain in concentration of the photon flux, reduces sensitivity and reduces the *z*-range over which measurements are useful.

The MVB indicated by equation (5.4) is marginally lower than that indicated by numerical analysis by Ram *et al*. [15], and processing of the images to obtain sharpness values cannot add extra information. However, the MVB is indicative of the least variance that the inversion of the data can yield, based on a model of the sensitivity of that data to changes in the parameter to be estimated. Differences in the model details used for evaluation of the MVB are potentially important here. However, the benefit of a formula that is easily evaluated for different experimental designs is substantial compared with a need to re-evaluate the MVB through numerical integration for each different case, provided that that formula gives satisfactory results.

## 6. Numerical simulations

The approximations used above deliver a simple formula for the MVB, but the validity of several of the approximations used has been justified only on heuristic grounds. For this reason, a series of simulations based on the numerically calculated intensity distribution in the images has been used to test the fidelity of equation (5.4). This is important to establish that the results of equation (5.4) can be used to optimize experimental systems for real-world compromises.

The numerical simulations were based on a diffraction-limited optical system in which the integral in equation (5.1) was evaluated to give the image intensity as a function of distance from the image centre. This intensity distribution was interpolated onto a Nyquist-sampled rectangular grid and the resulting ‘pixel intensities’ were used as the mean for the Poisson process associated with the photon count in each detector pixel. The total number of photons counted in any given ‘frame’ was also Poisson distributed. Simulated calibration data, in the form of 50 statistically independent ‘data frames’ generated with the same mean flux levels, were used to estimate 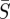 and 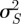 for ML algorithm PDF variation with source depth (details in the electronic supplementary material). The algorithm implemented to determine *z* from {*S*_1_, *S*_2_, *S*_3_} is the same as that used to analyse the experimental data. The ML solutions are shown as points and crosses in figure 4 for 768 detected photons distributed over the three images in each data frame and may be compared with the MVB estimations, from equation (5.4), with *α* = 2, in that figure.

**Figure 5.**
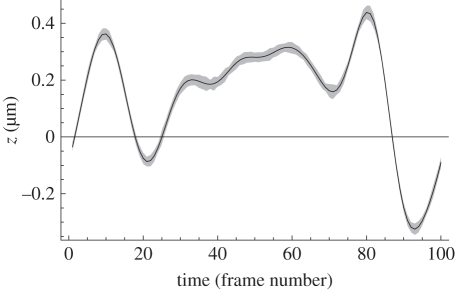
Actual position (line) and ML solution spread to the 1*σ* level (grey area) for a simulated movie of a particle on a smoothed random trajectory. The ‘time’ axis represents 100 simulated frames of data with an average of 768 detected photons per frame distributed between the three in-focus images (approx. 256 per image). A total of 50 movies were simulated and no smoothing has been applied to the solutions, so the grey area represents the scatter on the ML *z* estimates obtained from single data frames. In real measurements, and in the smoothed trajectory used in the simulation, the particle depth in consecutive frames must be correlated at some level, thus smoothing the time sequence of *z* estimates could have been used to reduce the spread in solutions. The unsmoothed variance from these movies is shown by the dots in figure 4.

Finally, as a test of the interpolations between calibration points in the ML algorithm, a ‘movie’ of an unresolved particle was simulated to provide ‘data frames’ corresponding to a smoothed pseudo-random motion in *z* over the range −0.33 µm < *z* < 0.44 µm. For each particle position, the image PSF was recalculated from equation (5.1), interpolated onto the detector pixel grid and the appropriate mean photon count per pixel evaluated. A series of 50 ‘movies’ was generated using these mean values to determine the photon count per pixel in each movie data frame. These data were provided blind to the individual (H.I.C.D.) doing the data inversion. The results for the ML estimation of the particle position in *z* are shown in figure 5, together with the true solution. No smoothing was applied to the frame-by-frame (i.e. time history) solutions from these movies, so the grey area is indicative of the instantaneous uncertainty in the *z*-estimate when only a single frame can be recorded on a fast-moving particle. The *z*-solution variance from these simulated measurements is 5 × 10^−4^ µm^2^ (s.d. 22 nm) compared with a prediction from equation (5.4) of 2 × 10^−4^ µm^2^ (10 nm). Smoothing over several data frames can improve the accuracy [3].

## 7. Results and discussion

The particle-tracking technique presented here has been developed with the objective of tracking objects such as vesicles [32] and single proteins [33] in live-cell microscopy. The algorithm requires two or more simultaneous images of the specimen that are focused on different depths within the specimen, and a simple, inexpensive optical attachment to capture three such images simultaneously from a standard biological microscope has been demonstrated.

Experimental results (figure 3) indicate that a depth accuracy of approximately 10 nm has been achieved on an IX71 microscope, using high flux levels with a monochromatic signal and a standard 12-bit CCD. These results, the MVB analysis and the numerical simulations presented here all indicate that the multi-focus sharpness technique can give depth resolution of 20 nm or better in samples greater than 4 µm thick, with realistic flux levels and with an experimentally simple system. Flux levels used in the experimental measurements are unknown but are probably greater than 10^6^ photons per image; however, the accuracy of depth reconstruction from these data is limited by the CCD bit depth. The resolutions obtained by this and similar techniques greatly exceed conventional resolution limits, but all such techniques exploit *a priori* information, and it has long been understood that this allows one to exceed conventional resolution criteria if a good SNR is available [34].

According to equation (5.4) a detected photon flux of approximately 3000 photons is required to deliver the 5 nm accuracy shown in vesicle tracking [3] and approximately 10^7^ detected photons is required to achieve the approximately 0.1 nm accuracy needed to resolve a base pair in molecular studies. This flux is easily compatible with that available in laser-tweezing measurements [35] and, for a given experimental requirement, there are four free parameters that can be manipulated to optimize the measurement system (detected flux, number and separation of the in-focus planes, NA of the objective).

However, fluorescent tags in live-cell biology generally provide strictly limited photon flux in order to minimize phototoxicity effects. Equation (5.4) suggests that approximately 10 nm accuracy can be achieved with count rates of approximately 1000 detected photons, indicating that the method will work with faint sources. These accuracies are comparable to those shown by Aguet *et al*. [9] and Ram *et al*. [15]; however, no detailed model of the imaging PSF is required in our approach. Interferometric techniques [11,12] by iPALM (interferometric photo-activated localization microscopy) offer a somewhat better accuracy, but the physically separate optical paths between specimen and interferometric beam combination mean that such techniques are challenging to implement without careful environmental control, including vibration isolation, and are probably not suitable for most microscopy laboratories. By comparison, the method discussed here, like [3,13,21], uses a simple system suitable for use with existing microscopes and cameras, not requiring stabilization and costing less than $5000 to build (most of which is the cost of packaging to exclude stray light—the DOEs cost $125 each). In the case of the double-helix method [21], a simple DOE can replace the more expensive spatial-light modulator and in many respects that optical scheme and the one described here differ principally in the DOE design. The DOE multi-focus technique described has already been realized using spatial light modulators [36].

Sharpness tracking does not depend on the use of a DOE to achieve multi-focus imaging—a system employing beam splitters or other approaches is equally capable of delivering the requisite multi-focus data with magnification or amplitude-splitting errors subsumed within the experimental calibration.

For application to depth measurement in image fields containing many particles, one must recognize that most techniques will have degraded performance if particle images overlap. Methods where this is an issue include PALM-based techniques [1,2,11], two-colour fluorescence in the context of single-molecule detection [37], the double-helix PSF [21] and the method described here. For all of these methods, the implications of the density of particles need to be considered. Image overlap might appear to be more of a problem with the deliberately defocused images used here, but, in most cases, at least two of the images are quite compact and the third image contributes relatively little to the *z*-determination but contributes to the depth range over which measurements can be made. In respect of the method described here, reducing the image-plane separation to the depth of field, i.e. *δ* approximately *a*, makes the images compact, virtually eliminates any overlap not already present in a diffraction-limited image of the specimen and extends the flux range over which the high-flux noise term dominates in equation (5.3)—these benefits are obtained at the expense of the range over which the particle depth may be accurately tracked. Reducing the image-plane separation below this value does not assist in controlling image overlap and sacrifices both accuracy and range for depth determination. The specific consequences of such compromises can be assessed using equation (5.4).

It may also be noted that the experimental data in figure 3 show unequal sharpness maxima in the different diffraction orders (background curves). The highest sharpness peak is consistently found to be associated with the in-focus image plane that is physically closest to the objective lens and the detected flux falls for sources positioned at greater depth. We thus attribute the variation in peak height principally to varying amounts of spherical aberration as a result of the immersion of the source in different depths of fluid. This highlights a particularly useful feature of the sharpness-based approach—one does not require a precise model of the optical system, the data reduction can be calibrated from experimental data, as was the case with the measurements presented here. Equation (5.3) provides a power law for scaling flux-dependent calibration PDF variance and it has been shown that this variance can be pragmatically tuned to accommodate instrumental drifts in order to obtain useful, if less accurate, depth tracking.

As noted elsewhere [15], few algorithms achieve the MVB, and we believe that further refinements in the probability models (e.g. to include both terms in equation (5.3)) will improve the depth accuracy shown in figures 3–5. In particular, such refinements may be expected significantly to improve algorithm performance at low flux and in the regions where the second term in equation (5.3) begins to dominate the noise. We believe that such improvements will ensure that the algorithm effectively achieves the calculated MVB.

To achieve optimum results will require the use of spatially resolving photon-counting cameras such as those developed within the Megaframe project (http://www.megaframe.eu/ (accessed on 18 December 2009)) and the use of such a CMOS camera offers the opportunity to subsume the sharpness calculation into the camera electronics for real-time application.

Finally, we note that the sharpness-ranging concept should be valid for application to extended incoherent source fields, either by collecting calibration data from similar-sized objects or by convolution of calibration data acquired on unresolved particles. In this respect, we note that the method shares a wavefront-sensing background with the ‘quantitative phase imaging’ approach (http://www.iatia.com.au/ (accessed on 18 December 2009)).

## References

[1] Betzig E. Patterson G. H. Sougrat R. Lindwasser O. W. Olenych S. Bonifacino J. S. Davidson M. W. Lippincott-Schwartz J. Hess H. F. 2006 Imaging intracellular fluorescent proteins at nanometer resolution. Science 313, 1642–1645 10.1126/science.1127344 (doi:10.1126/science.1127344) 16902090

[2] Juette M. F. Gould T. J. Lessard M. D. Mlodzianoski M. J. Nagpure B. S. Bennett B. T. Hess S. T. Bewersdorf J. 2008 Three-dimensional sub-100 nm resolution fluorescence microscopy of thick samples. Nat. Methods 5, 527–529 10.1038/nmeth.1211 (doi:10.1038/nmeth.1211) 18469823

[3] Watanabe T. M. Sato T. Gonda K. Higuchi H. 2007 Three-dimensional nanometry of vesicle transport in living cells using dual-focus imaging optics. Biochem. Biophys. Res. Commun. 359, 1–7 10.1016/j.bbrc.2007.04.168 (doi:10.1016/j.bbrc.2007.04.168) 17512495

[4] Qu X. H. Wu D. Mets L. Scherer N. F. 2004 Nanometer-localized multiple single-molecule fluorescence microscopy. Proc. Natl Acad. Sci. USA 101, 11 298–11 303 10.1073/pnas.0402155101 (doi:10.1073/pnas.0402155101) PMC509198 15277661

[5] Sbalzarini I. F. Koumoutsakos P. 2005 Feature point tracking and trajectory analysis for video imaging in cell biology. J. Struct. Biol. 151, 182–195 10.1016/j.jsb.2005.06.002 (doi:10.1016/j.jsb.2005.06.002) 16043363

[6] Poole D. R. Barenghi C. F. Sergeev Y. A. Vinen W. F. 2005 Motion of tracer particles in HeII. Phys. Rev. B 71, 064 514 10.1103/PhysRevB.71.064514 (doi:10.1103/PhysRevB.71.064514)

[7] Sheng J. Malkiel E. Katz J. 2006 Digital holographic microscope for measuring three-dimensional particle distributions and motions. Appl. Opt. 45, 3893–3901 10.1364/AO.45.003893 (doi:10.1364/AO.45.003893) 16724155

[8] Manz W. Arp G. Schumann-Kindel G. Szewzyk U. Reitner J. 2000 Widefield deconvolution epifluorescence microscopy combined with fluorescence in situ hybridization reveals the spatial arrangement of bacteria in sponge tissue. J. Microbiol. Methods 40, 125–134 10.1016/S0167-7012(99)00103-7 (doi:10.1016/S0167-7012(99)00103-7) 10699668

[9] Aguet F. Van De Ville D. Unser M. 2005 A maximum-likelihood formalism for sub-resolution axial localization of fluorescent nanoparticles. Opt. Expr. 13, 10 503–10 522 10.1364/OPEX.13.010503 (doi:10.1364/OPEX.13.010503) 19503266

[10] Ram S. Prabhat P. Ward E. S. Ober R. J. 2009 Improved single particle localization accuracy with dual objective multifocal plane microscopy. Opt. Expr. 17, 6881–6898 10.1364/OE.17.006881 (doi:10.1364/OE.17.006881) PMC2720637 19365515

[11] Shtengel G. 2009 Interferometric fluorescent super-resolution microscopy resolves 3D cellular ultrastructure. Proc. Natl Acad. Sci. USA 106, 3125–3130 10.1073/pnas.0813131106 (doi:10.1073/pnas.0813131106) 19202073 PMC2637278

[12] Von Middendorff C. Egner A. Geisler C. Hell S. W. Schonle A. 2008 Isotropic 3D nanoscopy based on single emitter switching. Opt. Expr. 16, 20 774–20 788 10.1364/OE.16.020774 (doi:10.1364/OE.16.020774) 19065216

[13] Mlodzianoski M. J. Juette M. F. Beane G. L. Bewersdorf J. 2009 Experimental characterization of 3D localization techniques for particle-tracking and super-resolution microscopy. Opt. Expr. 17, 8264–8277 10.1364/OE.17.008264 (doi:10.1364/OE.17.008264) 19434159

[14] Prabhat P. Ram S. Ward E. S. Ober R. J. 2004 Simultaneous imaging of different focal planes in fluorescence microscopy for the study of cellular dynamics in three dimensions. IEEE Trans. Nanobiosci. 3, 237–242 10.1109/TNB.2004.837899 (doi:10.1109/TNB.2004.837899) PMC2761735 15631134

[15] Ram S. Prabhat P. Chao J. Sally Ward E. Ober R. J. 2008 High accuracy 3D quantum dot tracking with multifocal plane microscopy for the study of fast intracellular dynamics in live cells. Biophys. J. 95, 6025–6043 10.1529/biophysj.108.140392 (doi:10.1529/biophysj.108.140392) 18835896 PMC2599831

[16] Toprak E. Balci H. Blehm B. H. Selvin P. R. 2007 Three-dimensional particle tracking via bifocal imaging. Nano Lett. 7, 2043–2045 10.1021/nl0709120 (doi:10.1021/nl0709120) 17583964

[17] Sun Y. McKenna J. D. Murray J. M. Ostap E. M. Goldman Y. E. 2009 Parallax: high accuracy three-dimensional single molecule tracking using split images. Nano Lett. 9, 2676–2682 10.1021/nl901129j (doi:10.1021/nl901129j) 19496608 PMC2728077

[18] Gobel W. Kampa B. M. Helmchen F. 2007 Imaging cellular network dynamics in three dimensions using fast 3D laser scanning. Nat. Methods 4, 73–79 10.1038/nmeth989 (doi:10.1038/nmeth989) 17143280

[19] Huang B. Wang W. Q. Bates M. Zhuang X. W. 2008 Three-dimensional super-resolution imaging by stochastic optical reconstruction microscopy. Science 319, 810–813 10.1126/science.1153529 (doi:10.1126/science.1153529) 18174397 PMC2633023

[20] Towers C. E. Towers D. P. Campbell H. I. Zhang S. Greenaway A. H. 2006 Three-dimensional particle imaging by wavefront sensing. Opt. Lett. 31, 1220–1222 10.1364/OL.31.001220 (doi:10.1364/OL.31.001220) 16642065

[21] Thompson M. A. Lew M. D. Badieirostami M. Moerner W. E. 2010 Localizing and tracking single nanoscale emitters in three dimensions with high spatiotemporal resolution using a double-helix point spread function. Nano Lett. 10, 211–218 10.1021/nl903295p (doi:10.1021/nl903295p) 20000821 PMC2806512

[22] Rosen J. Brooker G. 2008 Non-scanning motionless fluorescence three-dimensional holographic microscopy. Nat. Photonics 2, 190–195 10.1038/nphoton.2007.300 (doi:10.1038/nphoton.2007.300)

[23] Ikeda T. Popescu G. Dasari R. R. Feld M. S. 2005 Hilbert phase microscopy for investigating fast dynamics in transparent systems. Opt. Lett. 36, 1165–1167 10.1364/OL.30.001165 (doi:10.1364/OL.30.001165) 15945142

[24] Dalgarno P. A. Dalgarno H. I. C. Putoud A. Lambert R. Paterson L. Logan D. C. Towers D. P. Warburton R. J. Greenaway A. H. 2010 Multiplane imaging and three dimensional nanoscale particle tracking in biological microscopy. Opt. Expr. 18, 877–884 10.1364/OE.18.000877 (doi:10.1364/OE.18.000877) 20173908

[25] Blanchard P. M. Greenaway A. H. 1999 Simultaneous multiplane imaging with a distorted diffraction grating. Appl. Opt. 38, 6692–6699 10.1364/AO.38.006692 (doi:10.1364/AO.38.006692) 18324206

[26] Blanchard P. M. Greenaway A. H. 2000 Broadband simultaneous multiplane imaging. Opt. Commun. 183, 29–36 10.1016/S0030-4018(00)00874-9 (doi:10.1016/S0030-4018(00)00874-9)

[27] Djidel S. Gansel J. K. Campbell H. I. Greenaway A. H. 2006 High-speed, 3-dimensional, telecentric imaging. Opt. Expr. 14, 8269–8277 10.1364/OE.14.008269 (doi:10.1364/OE.14.008269) 19529202

[28] Greenaway A. 2010 Seeing more clearly. Phys. World 23, 33–36

[29] Muller R. A. Buffington A. 1974 Real-time correction of atmospherically degraded telescope images through image sharpening. J. Opt. Soc. Am. 64, 1200–1210 10.1364/JOSA.64.001200 (doi:10.1364/JOSA.64.001200)

[30] Aitken A. C. Sliverstone H. 1942 On the estimation of statistical parameters. Proc. R. Soc. Edinb. 61, 186–194

[31] Dainty J. C. Greenaway A. H. 1979 Estimation of spatial power spectra in speckle interferometry. J. Opt. Soc. Am. 69, 786–790 10.1364/JOSA.69.000786 (doi:10.1364/JOSA.69.000786)

[32] Racine V. Sachse M. Salamero J. Fraisier V. Trubuil A. Sibarita J.-B. 2007 Visualization and quantification of vesicle trafficking on a three-dimensional cytoskeleton network in living cells. J. Microsc. *(*Oxford*)* 225, 214–228 10.1111/j.1365-2818.2007.01723.x (doi:10.1111/j.1365-2818.2007.01723.x) 17371444

[33] Rickman C. Medine C. N. Dun A. R. Moulton D. J. Mandula O. Halemani N. D. Rizzoli S. O. Chamberlain L. H. Duncan R. R. 2010 t-SNARE protein conformations patterned by the lipid microenvironment. J. Biol. Chem. 285, 13 535–13 541 10.1074/jbc.M109.091058 (doi:10.1074/jbc.M109.091058) 20093362 PMC2859514

[34] Toraldo di Francia G. 1952 Super-gain antennas and optical resolving power. Suppl. Nuove Cimento 9, 426–435 10.1007/BF02903413 (doi:10.1007/BF02903413)

[35] Abbondanzieri E. A. Greenleaf W. J. Shaevitz J. W. Landick R. Block S. M. 2005 Direct observation of base-pair stepping by RNA polymerase. Nature 438, 460–465 10.1038/nature04268 (doi:10.1038/nature04268) 16284617 PMC1356566

[36] Maurer C. Khan S. Fassl S. Bernet S. Ritsch-Marte M. 2010 Depth of field multiplexing in microscopy. Opt. Expr. 18, 3023–3034 10.1364/OE.18.003023 (doi:10.1364/OE.18.003023) 20174133

[37] Agrawal A. Deo R. Wang G. D. Wang M. D. Nie S. 2008 Nanometer-scale mapping and single-molecule detection with color-coded nanoparticle probes. Proc. Natl Acad. Sci. USA 105, 3298–3303 10.1073/pnas.0712351105 (doi:10.1073/pnas.0712351105) 18305159 PMC2265145

